# Serum fibroblast growth factor 21 levels are related to subclinical atherosclerosis in patients with type 2 diabetes

**DOI:** 10.1186/s12933-015-0229-9

**Published:** 2015-06-06

**Authors:** Yang Xiao, Lingjiao Liu, Aimin Xu, Pengcheng Zhou, Zhaofeng Long, Yiting Tu, Xiaoyan Chen, Weili Tang, Gan Huang, Zhiguang Zhou

**Affiliations:** Key Laboratory of Diabetes Immunology, Ministry of Education, National Clinical Research Center for Metabolic Diseases, Diabetes Center, Second Xiangya Hospital, and Institute of Metabolism and Endocrinology, Central South University, Changsha, Hunan China; State Key Laboratory of Pharmaceutical Biotechnology, the University of Hong Kong, Hong Kong, China; Department of Medicine, the University of Hong Kong, Hong Kong, China; Department of Pharmacology and Pharmacy, the University of Hong Kong, Hong Kong, China; Research Center of Heart, Brain, Hormone, and Healthy Aging, the University of Hong Kong, Hong Kong, China; Department of Geriatrics, Second Xiangya Hospital, Central South University, Changsha, Hunan China

**Keywords:** Fibroblast growth factor 21, Type 2 diabetes, Subclinical atherosclerosis, Intima-media thickness

## Abstract

**Background:**

Fibroblast growth factor 21 (FGF21), a glucose and lipid metabolic regulator, has recently been demonstrated to be associated with cardiovascular diseases (CVD) such as carotid atherosclerosis, coronary heart disease and carotid artery plaques. However, the relationship between circulating FGF21 and subclinical atherosclerosis or atherosclerosis of other arteries such as the femoral and iliac artery remains unclear. In this study, we evaluated the association of serum FGF21 with intima-media thickness (IMT) and subclinical atherosclerosis in type 2 diabetic patients.

**Methods:**

Serum FGF21 levels were detected by enzyme-linked immunosorbent assay in 212 newly diagnosed type 2 diabetic patients without clinical symptoms of atherosclerosis or cardiovascular diseases. IMT of the carotid, femoral, and iliac arteries were measured by high-resolution B-mode ultrasound to determine the presence of subclinical atherosclerosis, which was defined as having an IMT > 1.0 mm and/or plaque on one or more of the three arteries without any clinical manifestations. The relationship between serum FGF21 levels and subclinical atherosclerosis was analyzed.

**Results:**

Serum FGF21 levels were significantly higher in patients with subclinical atherosclerosis compared to those without [261.3 (135.1–396.4) versus 144.9 (95.9–223.0) ng/L, *P* < 0.001]. These differences were also observed in both men and women with subclinical atherosclerosis compared to their respective groups without [men: 243.2 (107.6–337.0) versus 136.8 (83.6–212.8) ng/L, *P* = 0.048; women: 292.4 (174.2–419.9) versus 160.4 (115.3–258.5) ng/L, *P* = 0.001]. Moreover, serum FGF21 levels showed a significantly positive correlation with carotid IMT in women (r = 0.23, *P* = 0.018) and with iliac IMT in both genders (women: r = 0.27, *P* = 0.005; men: r = 0.22, *P* = 0.024). Multiple logistic regression analysis further showed that serum FGF21 was an independent impact factor for subclinical atherosclerosis in patients with type 2 diabetes.

**Conclusions:**

Serum FGF21 is elevated in newly diagnosed type 2 diabetes, and positively correlates with carotid and iliac lesions in patients with subclinical atherosclerosis, especially in women. High levels of FGF21 may be a compensatory reaction to offset atherosclerosis.

## Background

Fibroblast growth factor 21 (FGF21) is a member of the endocrine FGF subfamily, which also includes FGF19 and FGF23 [1]. Unlike classical FGF molecules that act in an autocrine manner, these three members are present in the circulation and possess hormone-like activities [2]. A growing body of evidence demonstrates that FGF21 is an important metabolic regulator with multiple beneficial effects on glucose and lipid homeostasis in animals [3, 4]. Systemic administration of recombinant FGF21 reduced plasma glucose and lipids to near normal levels in both ob/ob and db/db mouse models as well as diabetic monkeys [3, 4]. Likewise, FGF21 transgenic mice rendered protection against diet-induced obesity and metabolic disorders [4].

Despite the multiple salutatory effects of FGF21 on glucose and lipid metabolism in animals, circulating FGF21 levels are elevated in obese subjects [5, 6] and patients with impaired glucose tolerance [7], type 2 diabetes mellitus [5, 7, 8], dyslipidemia [9], and nonalcoholic fatty liver disease (NAFLD) [10, 11]. Additionally, circulating FGF21 levels are positively associated with body adiposity index, insulin resistance [6], hepatic lipid levels [11], and markers of liver injury [9]. Thus, it has been proposed that the elevated level of FGF21 (a condition termed “hyper-FGF21-nemia”) in obese subjects is due to a decrease in the number of responsive FGF21 receptors on target cells, a phenomenon known as FGF21 resistance [12].

Recently, several reports have examined the role of FGF21 in cardiovascular diseases (CVD) in humans. Elevated serum FGF21 levels have been shown in subjects with coronary heart disease (CHD) [13, 14] and was associated with both carotid atherosclerosis in women and carotid artery plaques in type 2 diabetes subjects [15, 16]. However, circulating FGF21 levels in type 2 diabetic patients with or without subclinical atherosclerosis or its association with atherosclerosis of other arteries such as the femoral and iliac artery remain unclear. In this study, we assessed the relationship between circulating FGF21 and carotid, femoral and iliac intima-media thickness (IMT) as well as subclinical atherosclerosis in patients with newly diagnosed type 2 diabetes.

## Methods

### Participants

Our study consisted of 212 patients who were diagnosed within 1 year with type 2 diabetes and recruited from the Diabetes Center, the Second Xiangya Hospital, Central South University. All patients were enrolled in the Chinese National Tenth and Eleventh Five Tackling Key Project from 2002 to 2007. Type 2 diabetes was diagnosed according to the American Diabetes Association criteria [17]. Exclusion criteria were clinical symptoms of atherosclerosis; presence of CVD (a history of physician-diagnosed myocardial infarction, angina, heart failure, stroke, or transient ischemic attack or who had undergone an invasive cardiovascular procedure); diabetic nephropathy or diabetic retinopathy; or severe liver and kidney dysfunction.

Hypertension was defined as having a blood pressure of ≥140/90 mm Hg, or if the patient was regularly taking antihypertensive medications. Dyslipidemia was diagnosed as having one or more of the following criteria: (1) fasting triglyceride (TG) ≥ 1.7 mmol/L; (2) high-density lipoprotein-cholesterol (HDL-c) < 1.30 mmol/L in female and < 1.0 mmol/L in male; (3) low-density lipoprotein-cholesterol (LDL-c) ≥ 3.4 mmol/L; (4) already on lipid-lowering drug according to the United States Adult Treatment Panel III [18]. Central obesity was defined as a waist circumference (WC) >90 cm for men, >80 cm for women. Smoking referred to both current and past smokers, whereas nonsmoker referred to those who never smoked.

This study was performed with the approval of the Institutional Review Board of the Second Xiangya Hospital, Central South University, and written informed consent was obtained from each patient.

### Physical and biochemical assessment

All patients were assessed after an overnight fasting. Anthropometric measurements including height, body weight, body mass index (BMI), WC and blood pressure were taken. BMI was calculated as weight divided by squared-height (kg/m^2^). Blood pressure was measured on the right arm with the patient seated for at least 10 min. WC was measured midway between the lower rib margin and iliac crest. Detailed family histories, including history of CVD, were obtained and evaluated from a questionnaire. Blood was drawn for fasting glucose, insulin, cholesterol, TG and hemoglobin A1C (HbA1c) levels. Plasma glucose, serum cholesterol, and TG levels were measured enzymatically. Serum insulin was detected using chemiluminescent immunoassays. HbA1c was determined by an ion exchange HPLC method. A human FGF21 enzyme-linked immunosorbent assay (ELISA) kit established in our laboratory (Antibody and Immunoassay Services, the University of Hong Kong) was used to determine serum FGF21 levels [8]. The intra- and inter-assay coefficients of variance were 4.0–5.0 % and 3.5–10.2 %, respectively.

### Vascular ultrasound measurement

The IMT of common carotid, femoral and common iliac arteries on the right side were evaluated by high-resolution B-mode ultrasound (128XP/10 system; Acuson, Mountain View, California, USA). The measurements of IMT were made at the site of greatest thickness. Plaque was defined as having an IMT ≥1.3 mm or a focal protrusion into the lumen with a thickness of at least 50 % more than the adjacent intima-media complex. The definition of subclinical atherosclerosis was having an IMT > 1.0 mm and/or plaque on one or more of the three arteries without any clinical manifestations [19, 20].

### Statistical analysis

All statistical analyses were performed with Statistical Package for Social Science Version 16.0 (SPSS 16.0, Inc., Chicago, IL). Data were expressed as a mean ± SD or median with interquartile range, as appropriate. Data that were not normally distributed, as determined using the Kolmogorov-Smirnov test, were logarithmically transformed before analysis. Correlations between FGF21 and biochemical variables were analyzed with Pearson correlation. Comparisons between groups were performed using *χ*^2^ tests for categorical variables and independent-samples *T* test for continuous variables as indicated. Multiple logistic regression analysis was done to determine independent factors of subclinical atherosclerosis, and included variables which were biologically likely to be related with atherosclerotic status. Two-sided *P* values <0.05 were considered statistically significant.

## Results

The physical and biochemical characteristics of all type 2 diabetic patients including FGF21 levels, IMT, and cardiovascular risks are summarized in Table 1. Compared with women, men had similar percentage of subclinical atherosclerosis and FGF21. For the established cardiovascular risk factors, men had higher WC, serum TG, and percentage of current or past smokers, but a lower HDL-c level compared to women (for WC and TG, *P* = 0.001; for smokers and HDL-c, *P* < 0.001). In addition, women were older and had higher systolic blood pressure (*P* = 0.011 and *P* = 0.046, respectively).

**Table 1:** Clinical and biochemical characteristics of the study patients

Parameters	All patients (*n* = 212)	Men (*n* = 107)	Women (*n* = 105)	*P* Value (Men vs Women)
Age, year	54.1 ± 8.5	52.6 ± 9.7	55.6 ± 6.9	0.011
Family history of cardiovascular diseases, %	45.8	43.9	47.6	0.589
Current/past smoker, %	38.2	70.1	5.7	<0.001
Body mass index, kg/m^2^	24.5 ± 2.8	24.9 ± 2.7	24.2 ± 2.8	0.069
Waist circumference, cm	87.2 ± 8.2	89.1 ± 8.2	85.2 ± 7.8	0.001
Central obesity, %	52.8	34.6	71.4	<0.001
Fasting glucose, mmol/L	7.8 ± 3.3	7.6 ± 2.5	8.1 ± 3.9	0.236
HbA1c, %	7.7 ± 2.4	7.8 ± 2.3	7.7 ± 2.5	0.736
Fasting insulin^a^, mIU/L	13.3 (9.0–19.0)	14.0 (9.3–18.0)	12.1 (9.0–21.0)	0.806
Systolic blood pressure, mm Hg	119.1 ± 17.2	116.7 ± 15.6	121.5 ± 18.5	0.046
Diastolic blood pressure, mm Hg	76.6 ± 10.7	77.1 ± 10.4	76.1 ± 11.0	0.488
Hypertension, %	23.1	19.6	26.7	0.224
Triglycerides^a^, mmol/L	1.62 (1.16–2.53)	1.87 (1.24–3.31)	1.51 (1.11–2.07)	0.001
Low-density lipoprotein-cholesterol, mmol/L	3.1 ± 1.0	3.0 ± 1.1	3.1 ± 0.9	0.501
High-density lipoprotein-cholesterol, mmol/L	1.3 ± 0.4	1.2 ± 0.4	1.4 ± 0.3	<0.001
Dyslipidemia, %	73.1	72.9	73.3	0.943
Carotid intima-media thickness, mm	0.75 ± 0.20	0.75 ± 0.19	0.75 ± 0.21	0.955
Femoral intima-media thickness, mm	0.77 ± 0.23	0.75 ± 0.18	0.79 ± 0.27	0.217
Iliac intima-media thickness, mm	0.85 ± 0.22	0.85 ± 0.23	0.85 ± 0.21	0.953
Subclinical atherosclerosis, %	20.8	19.6	21.9	0.683
Fibroblast growth factor 21^a^, ng/L	154.0 (105.6–283.3)	142.4 (86.1–269.8)	175.9 (125.1–309.0)	0.137

Data presented as mean ± SD or median (interquartile range)

^a^Log-transformed before analysis

**Fig. 1:**
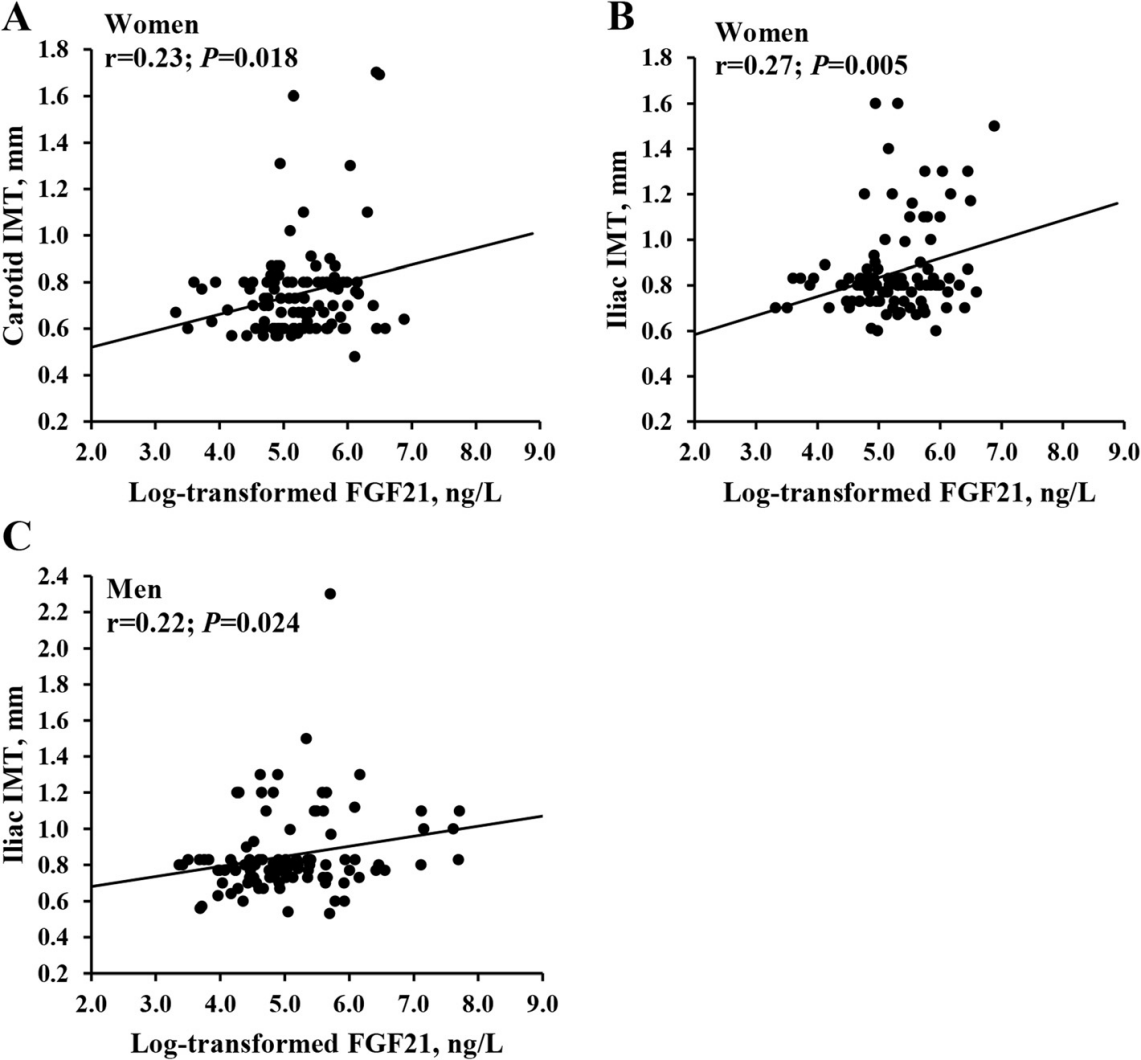
Correlations between FGF21 levels and IMT. Correlations between serum FGF21 levels and carotid IMT (**a**, *n* = 105) or iliac IMT (**b**, *n* = 105) in women with type 2 diabetes; correlation between serum FGF21 levels and iliac IMT (**c**, *n* = 107) in men with type 2 diabetes

**Fig. 2:**
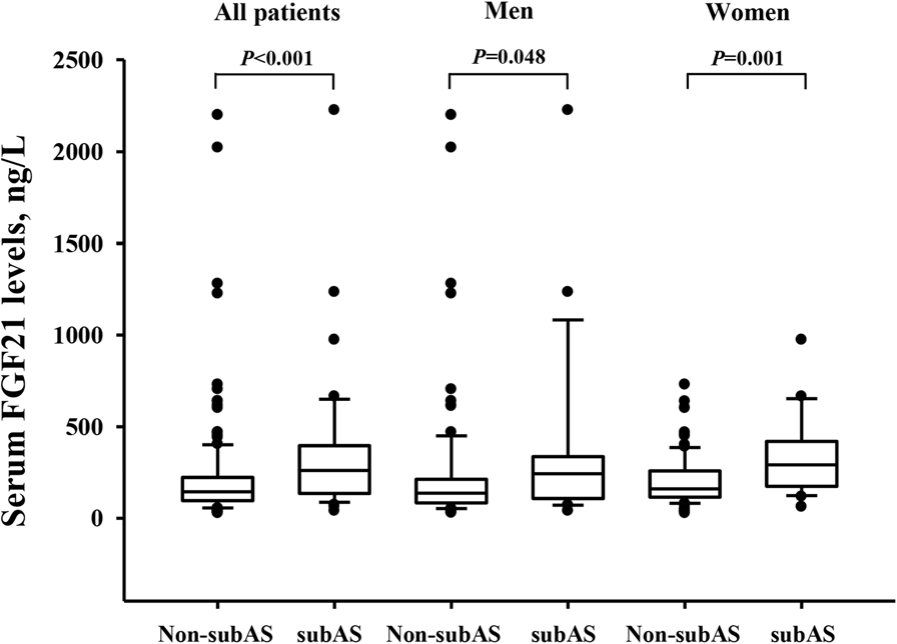
Serum FGF21 levels in type 2 diabetic patients with subclinical atherosclerosis or without subclinical atherosclerosis. Serum levels of FGF21 in patients with subclinical atherosclerosis (subAS) or patients without subclinical atherosclerosis (non-subAS) in the entire cohort, men or women subgroup are shown as box-and-whisker plots. The horizontal line in the middle of each box indicates the median value; the top and bottom borders of the boxes represent the 75th and 25th percentiles, respectively; the whiskers represent the 10th and 90th percentiles, respectively; and the dots represent the outliers

In men, serum FGF21 levels positively correlated with fasting glucose and TG (*P* = 0.034 and *P* = 0.042, respectively, Table 2). In women, serum FGF21 levels correlated positively with WC, fasting glucose and TG (P = 0.033, *P* = 0.035 and *P* = 0.024, respectively). FGF21 levels also showed a significantly positive correlation with carotid IMT in women (r = 0.23, *P* = 0.018, Fig. 1a) as well as iliac IMT in both genders (women: r = 0.27, *P* = 0.005, Table 1, Fig. 1b; men: r = 0.22, *P* = 0.024, Fig. 1c). Furthermore, serum FGF21 levels were strikingly higher in patients with subclinical atherosclerosis compared to patients without subclinical atherosclerosis [261.3 (135.1–396.4) versus 144.9 (95.9–223.0) ng/L, *P* < 0.001]. These differences were observed for both genders with subclinical atherosclerosis compared to their respective groups without [men: 243.2 (107.6–337.0) versus 136.8 (83.6–212.8) ng/L, *P* = 0.048; women: 292.4 (174.2–419.9) versus 160.4 (115.3–258.5) ng/L, *P* = 0.001, Fig. 2].

**Table 2:** Correlations of Serum FGF21 levels with various clinical and biochemical parameters

	Men (*n* = 107)		Women (*n* = 105)	
	r	*P* value	r	*P* value
Age, year	0.10	0.302	0.15	0.129
Body mass index, kg/m^2^	−0.01	0.930	−0.02	0.848
Waist circumference, cm	0.03	0.780	0.21	0.033
Systolic blood pressure, mmHg	0.07	0.457	0.04	0.694
Diastolic blood pressure, mmHg	0.03	0.776	−0.04	0.699
Fasting glucose, mmol/L	0.21	0.034	0.21	0.035
HbA1c, %	−0.11	0.258	0.03	0.738
Fasting insulin^a^, mIU/L	−0.11	0.254	0.04	0.691
High-density lipoprotein-cholesterol, mmol/L	0.10	0.324	−0.01	0.919
Low-density lipoprotein-cholesterol, mmol/L	0.14	0.165	0.04	0.716
Triglycerides^a^	0.20	0.042	0.22	0.024
Carotid intima-media thickness, mm	0.00	0.997	0.23	0.018
Femoral intima-media thickness, mm	0.02	0.872	0.15	0.132
Iliac intima-media thickness, mm	0.22	0.024	0.27	0.005

^a^Log-transformed before analyses

On a sex-specific multiple logistic regression model, independent, significant impact factors for subclinical atherosclerosis in both genders were identified: for men these included age, BMI, smoking history, family history of CVD, fasting glucose, fasting insulin, hypertension, dyslipidemia and serum FGF21; for women these included age, BMI, smoking history, family history of CVD, menopausal status, fasting glucose, fasting insulin, hypertension, dyslipidemia and serum FGF21 (Model 1, Table 3). Elevated serum FGF21 was found to be an independent impact factor for subclinical atherosclerosis in the full regression model, in which all relevant confounding variables were entered for both men and women (*P* = 0.014 and *P* = 0.029, respectively). Family history of CVD and BMI were also independently associated with subclinical atherosclerosis in men (*P* = 0.004 and *P* = 0.036, respectively), while age and family history of CVD were independently associated with subclinical atherosclerosis in women (*P* = 0.021 and *P* = 0.034, respectively). If BMI was replaced by WC in models of both genders (Model 2), FGF21 remained as a significant impact factor associated with subclinical atherosclerosis in type 2 diabetes in both men and women (*P* = 0.026 and *P* = 0.037, respectively).

**Table 3:** Multiple logistic regression analysis showing the parameters with significant independent associations with subclinical atherosclerosis

	Men (*n* = 107)				Women (*n* = 105)			
	Model 1		Model 2		Model 1		Model 2	
Parameters	OR(95 % CI)	*P* value	OR(95 % CI)	*P* value	OR(95 % CI)	*P* value	OR(95 % CI)	*P* value
Age	1.01(0.95–1.07)	0.828	0.99(0.93–1.05)	0.718	1.17(1.02–1.33)	0.021	1.15(1.01–1.30)	0.040
Smoking	1.07(0.30–3.73)	0.921	1.25(0.38–4.15)	0.715	3.68(0.13–104.52)	0.455	4.23(0.18–97.23)	0.368
Family history of cardiovascular diseases	6.411(1.82–22.56)	0.004	4.18(1.31–13.35)	0.016	4.02(1.11–14.55)	0.034	4.16(1.16–14.98)	0.029
Menopause status	-	-	-	-	1.24(0.08–19.68)	0.877	1.39(0.08–23.30)	0.818
Body mass index	1.27(1.02–1.59)	0.036	-	-	1.18(0.90–1.55)	0.235	-	-
Waist circumference	-	-	0.98(0.90–1.05)	0.540	-	-	1.02(0.95–1.09)	0.661
Hypertension	0.82(0.19–3.50)	0.790	1.11(0.28–4.43)	0.887	0.59(0.15–2.36)	0.459	0.63(0.16–2.49)	0.510
Dyslipidemia	0.35(0.10–1.22)	0.100	0.38(0.11–1.28)	0.117	3.36(0.71–15.77)	0.125	3.18(0.70–14.53)	0.135
Fasting glucose	0.91(0.72–1.15)	0.435	0.96(0.78–1.19)	0.714	1.04(0.91–1.19)	0.583	1.03(0.90–1.17)	0.677
Fasting insulin^a^	0.46(0.15–1.43)	0.181	0.79(0.28–2.25)	0.656	0.63(0.20–1.96)	0.420	0.87(0.32–2.34)	0.781
Fibroblast growth factor 21^a^	2.20(1.17–4.13)	0.014	1.98(1.09–3.60)	0.026	3.08(1.12–8.42)	0.029	2.90(1.07–7.86)	0.037

^a^Log-transformed before analyses

## Discussion

In the current study we report for the first time that serum FGF21 levels are strikingly elevated in newly diagnosed type 2 diabetic patients with subclinical atherosclerosis compared to those without subclinical atherosclerosis. This observation remained true in both men and women when the results were analyzed independently. Moreover, we detected a strong association of FGF21 with carotid IMT in women as well as iliac IMT in men and women with type 2 diabetes.

Several risk factors have been proposed as potential markers for improved detection of subclinical atherosclerosis. In particular, physical measurements such as neck circumference [21] and abdominal adiposity [22]; inflammatory biomarkers such as C-reactive protein (CRP) [23]; lipid parameters such as oxidized LDL; adhesion molecules such as E-selectin; cytokines such as osteoprotegerin; and adipokines such as leptin [24], have all been associated with subclinical atherosclerosis in different ethnic groups. However, each of these factors provides only a rather limited information for cardiovascular risk. Therefore, identification of additional risk factors and/or biomarkers for atherosclerosis may help a better understanding of the disease, which in turn facilitate early diagnosis.

Recently, more studies have indicated the relevance of FGF21 to CVD in humans. Chow et al. found that elevated serum FGF21 levels were associated with carotid IMT independent of cardiovascular risk factors [15]. In separate group studies, Lin et al. and Shen et al. examined patients using diagnostic criteria and coronary arteriography, respectively, to show that serum FGF21 levels were significantly higher in CHD patients compared to healthy controls [13, 14]. An et al. showed that type 2 diabetic subjects with carotid artery plaques expressed higher levels of FGF21 than those without plaque [16]. Arterial stiffness, as measured by brachial-ankle pulse wave velocity, has also been reported to be related to FGF21 levels in obese women [25]. Surprisingly, the association between arterial stiffness and FGF21 was diminished by a 3 month exercise program, suggesting that hyper-FGF21-nemia is reversible. In addition, the presence of higher serum FGF21 was associated with a significant increase in combined cardiovascular morbidity and mortality [26]. Our study, which is focused on the early stage of atherosclerosis in newly diagnosed type 2 diabetes patients, suggests that FGF21 is linked to the initiation and formation of atherosclerosis.

The role of FGF21 in atherosclerosis has been highlighted recently. FGF21 was found to be upregulated and secreted from hepatocytes and adipocytes into the circulation to protect ischemic cardiomyocytes [27]. FGF21 is also expressed in endothelial cells and can be stimulated by high glucose, which in turn protects against cellular damage and endothelial nitric oxide synthase (eNOS) dysfunction in endothelial cells [28]. Therefore, it is possible that in different tissues FGF21 expression and secretion is induced by atherogenic factors as a defense response to counteract the disease. FGF21 may mediate its anti-atherosclerotic effects via induction of adiponectin in adipocytes and suppression of cholesterol synthesis in hepatocytes [29]. However, the protective effects of FGF21 may be inhibited due to downregulation of its co-receptor β-Klotho caused by TNF-α in the chronic inflammatory state, leading to FGF21 resistance [30]. The presence of elevated FGF21 levels in patients with subclinical atherosclerosis may also be attributed to a compensatory response, secondary to FGF21 resistance.

In this study, serum FGF21 was associated with carotid and iliac IMT in women but only iliac IMT in men. A previous study also showed gender-specific differences in the association between serum FGF21 and carotid IMT [15]. One explanation for this is the difference in risk factors between men and women in this study. As male participants in our study had a higher prevalence of smoking and higher serum levels of TG, the impact of FGF21 on carotid atherosclerosis might be outweighed by these confounding risk factors. Another explanation might be the contribution of sex hormones to atherosclerosis. One study showed a negative correlation of serum FGF21 and estradiol which can protect premenopausal women from CVD [31]. The different mechanism(s) underlying the association of FGF21 and sex hormones with respect to CVD remain to be established. In addition, we found a positive correlation between serum FGF21 with iliac IMT but not with femoral IMT in both genders, which suggests that there may be some differences in the pathogenesis of atherosclerosis in these 2 vessels even though they are nearby. It is possible that shear stress applied to the femoral artery might be different from that applied to the common iliac artery because of the vascular curvature existing at the femoral site, which may lead to disturbed blood flow that influences atherogenesis [32].

In line with previous findings [13, 15], we found that serum FGF21 levels were positively correlated to TG and fasting glucose. We failed to observe a significant association between FGF21 and fasting insulin, which has been proven by other authors [9, 33], suggesting that FGF21 might play a role in glucose metabolism but not through insulin secretion or actions. Both fasting glucose and postprandial glucose levels contribute to HbA1c, however there was no significant correlation between FGF21 and 2 h-postprandial glucose (data not shown), which is a possible reason for the lack of association between FGF21 and HbA1c.

Intriguingly, the association between serum FGF21 and WC was only present in women, which was consistent with another study [15]. A reason for this may be that in females, not only visceral adipose accumulation, but also subcutaneous adipose accumulation contributes to WC. FGF21 mRNA expression was shown to be 1-fold higher in subcutaneous adipose tissue than in visceral adipose tissue in subjects with a BMI < 40 kg/m^2^, whereas in subjects with a BMI > 40 kg/m^2^, FGF21 mRNA expression was similar in both tissues [5]. This suggests that the production of FGF21 from subcutaneous adipose tissue is a more important source for elevated serum FGF21, with the exception of the liver.

The median of serum FGF21 in the current study was different from our previous work both of which recruited type 2 diabetic patients but quite differed in sample size [8]. Interindividual variation in FGF21 levels may partially account for this difference since it has been reported that even healthy individuals show a wide range in serum FGF21 (21–5300 ng/L) [34]. Another reason is that given the non-normal distribution of serum FGF21 levels, we can only use the median which is less sensitive and efficient than the mean to describe the data.

### Limitations

Our study has several limitations. First, our present study is limited by the lack of measurements for NAFLD. Several studies have shown that FGF21 is increased in NAFLD [10, 11]. It has also been reported that liver fat is the only measure of adiposity that influences circulating FGF21 levels [35]. In addition, another study found that subjects with coronary artery disease had significantly higher serum FGF21 when the presence of NAFLD was taken into account [14]. Therefore, further studies will be needed to elucidate the impact of NAFLD on the association of FGF21 and subclinical atherosclerosis. Second, the current study population included only subjects with type 2 diabetes, therefore our findings may not be directly applicable to the general population because of the sampling bias.

## Conclusions

In our study, we have shown that serum FGF21 is elevated in newly diagnosed type 2 diabetes, and positively correlates with carotid and iliac lesions in patients with subclinical atherosclerosis, especially in women. Indeed, high levels of FGF21 may be a compensatory reaction to offset atherosclerosis, suggesting that FGF21 might be a potential therapeutic target for this disease.

## Abbreviations

BMI: Body mass index
CHD: Coronary heart disease
CRP: C-reactive protein
CVD: Cardiovascular diseases
ELISA: Enzyme-linked immunosorbent assay
eNOS: Endothelial nitric oxide synthase
FGF21: Fibroblast growth factor 21
HbA1c: Hemoglobin A1c
HDL-c: High-density lipoprotein-cholesterol
HT: Hypertension
IMT: Intima-media thickness
LDL-c: Low-density lipoprotein-cholesterol
NAFLD: Non-alcoholic fatty liver disease
subAS: Subclinical atherosclerosis
TG: Triglyceride
WC: Waist circumference

## References

[1] Itoh N, Ornitz DM (2011). Fibroblast growth factors: from molecular evolution to roles in development, metabolism and disease. J Biochem.

[2] Itoh N, Ornitz DM (2008). Functional evolutionary history of the mouse Fgf gene family. Dev Dyn.

[3] Kharitonenkov A, Wroblewski VJ, Koester A, Chen YF, Clutinger CK, Tigno XT (2007). The metabolic state of diabetic monkeys is regulated by fibroblast growth factor-21. Endocrinology.

[4] Kharitonenkov A, Shiyanova TL, Koester A, Ford AM, Micanovic R, Galbreath EJ (2005). FGF-21 as a novel metabolic regulator. J Clin Invest.

[5] Mraz M, Bartlova M, Lacinova Z, Michalsky D, Kasalicky M, Haluzikova D (2009). Serum concentrations and tissue expression of a novel endocrine regulator fibroblast growth factor-21 in patients with type 2 diabetes and obesity. Clin Endocrinol (Oxf).

[6] Zhang X, Yeung DC, Karpisek M, Stejskal D, Zhou ZG, Liu F (2008). Serum FGF21 levels are increased in obesity and are independently associated with the metabolic syndrome in humans. Diabetes.

[7] Chavez AO, Molina-Carrion M, Abdul-Ghani MA, Folli F, Defronzo RA, Tripathy D (2009). Circulating fibroblast growth factor-21 is elevated in impaired glucose tolerance and type 2 diabetes and correlates with muscle and hepatic insulin resistance. Diabetes Care.

[8] Xiao Y, Xu A, Law LS, Chen C, Li H, Li X (2012). Distinct changes in serum fibroblast growth factor 21 levels in different subtypes of diabetes. J Clin Endocrinol Metab.

[9] Li H, Bao Y, Xu A, Pan X, Lu J, Wu H (2009). Serum fibroblast growth factor 21 is associated with adverse lipid profiles and gamma-glutamyltransferase but not insulin sensitivity in Chinese subjects. J Clin Endocrinol Metab.

[10] Yilmaz Y, Eren F, Yonal O, Kurt R, Aktas B, Celikel CA (2010). Increased serum FGF21 levels in patients with nonalcoholic fatty liver disease. Eur J Clin Invest.

[11] Li H, Fang Q, Gao F, Fan J, Zhou J, Wang X (2010). Fibroblast growth factor 21 levels are increased in nonalcoholic fatty liver disease patients and are correlated with hepatic triglyceride. J Hepatol.

[12] Fisher FM, Chui PC, Antonellis PJ, Bina HA, Kharitonenkov A, Flier JS (2010). Obesity is a fibroblast growth factor 21 (FGF21)-resistant state. Diabetes.

[13] Lin Z, Wu Z, Yin X, Liu Y, Yan X, Lin S (2010). Serum levels of FGF-21 are increased in coronary heart disease patients and are independently associated with adverse lipid profile. PLoS One.

[14] Shen Y, Ma X, Zhou J, Pan X, Hao Y, Zhou M (2013). Additive relationship between serum fibroblast growth factor 21 level and coronary artery disease. Cardiovasc Diabetol.

[15] Chow WS, Xu A, Woo YC, Tso AW, Cheung SC, Fong CH (2013). Serum fibroblast growth factor-21 levels are associated with carotid atherosclerosis independent of established cardiovascular risk factors. Arterioscler Thromb Vasc Biol.

[16] An SY, Lee MS, Yi SA, Ha ES, Han SJ, Kim HJ (2012). Serum fibroblast growth factor 21 was elevated in subjects with type 2 diabetes mellitus and was associated with the presence of carotid artery plaques. Diabetes Res Clin Pract.

[17] American Diabetes Association (2007). Diagnosis and classification of diabetes mellitus. Diabetes Care.

[18] Expert Panel on Detection E (2001). Treatment of high blood cholesterol in a: executive summary of the third report of the national cholesterol education program (NCEP) expert panel on detection, evaluation, and treatment of high blood cholesterol in adults (adult treatment panel III). Jama.

[19] Kardys I, Oei HH, van der Meer IM, Hofman A, Breteler MM, Witteman JC (2006). Lipoprotein-associated phospholipase A2 and measures of extracoronary atherosclerosis: the Rotterdam Study. Arterioscler Thromb Vasc Biol.

[20] Balbarini A, Buttitta F, Limbruno U, Petronio AS, Baglini R, Strata G (2000). Usefulness of carotid intima-media thickness measurement and peripheral B-mode ultrasound scan in the clinical screening of patients with coronary artery disease. Angiology.

[21] Liang J, Wang Y, Li H, Liu X, Qiu Q, Qi L (2014). Neck circumference and early stage atherosclerosis: the cardiometabolic risk in Chinese (CRC) study. Cardiovasc Diabetol.

[22] Lukich A, Gavish D, Shargorodsky M (2014). Normal weight diabetic patients versus obese diabetics: relation of overall and abdominal adiposity to vascular health. Cardiovasc Diabetol.

[23] Ridker PM, Stampfer MJ, Rifai N (2001). Novel risk factors for systemic atherosclerosis: a comparison of C-reactive protein, fibrinogen, homocysteine, lipoprotein(a), and standard cholesterol screening as predictors of peripheral arterial disease. JAMA.

[24] Reddy RK, Mahendra J, Gurumurthy P, Jayamathi Babu S (2015). Identification of predictable biomarkers in conjunction to Framingham risk score to predict the risk for cardiovascular disease (CVD) in Non cardiac subjects. J Clin Diagn Res.

[25] Yang SJ, Hong HC, Choi HY, Yoo HJ, Cho GJ, Hwang TG (2011). Effects of a 3 month combined exercise programme on fibroblast growth factor 21 and fetuin-A levels and arterial stiffness in obese women. Clin Endocrinol (Oxf).

[26] Lenart-Lipinska M, Matyjaszek-Matuszek B, Gernand W, Nowakowski A, Solski J (2013). Serum fibroblast growth factor 21 is predictive of combined cardiovascular morbidity and mortality in patients with type 2 diabetes at a relatively short-term follow-up. Diabetes Res Clin Pract.

[27] Liu SQ, Roberts D, Kharitonenkov A, Zhang B, Hanson SM, Li YC (2013). Endocrine protection of ischemic myocardium by FGF21 from the liver and adipose tissue. Sci Rep.

[28] Wang XM, Song SS, Xiao H, Gao P, Li XJ, Si LY (2014). Fibroblast growth factor 21 protects against high glucose induced cellular damage and dysfunction of endothelial nitric-oxide synthase in endothelial cells. Cell Physiol Biochem.

[29] Lin Z, Pan X, Wu F, Ye D, Zhang Y, Wang Y (2015). Fibroblast Growth Factor 21 Prevents Atherosclerosis by Suppression of Hepatic Sterol Regulatory Element-Binding Protein-2 and Induction of Adiponectin in Mice. Circulation.

[30] Diaz-Delfin J, Hondares E, Iglesias R, Giralt M, Caelles C, Villarroya F (2012). TNF-alpha represses beta-Klotho expression and impairs FGF21 action in adipose cells: involvement of JNK1 in the FGF21 pathway. Endocrinology.

[31] Zhang X, Hu Y, Zeng H, Li L, Zhao J, Zhao J (2015). Serum fibroblast growth factor 21 levels is associated with lower extremity atherosclerotic disease in Chinese female diabetic patients. Cardiovasc Diabetol.

[32] Smedby O (1998). Geometrical risk factors for atherosclerosis in the femoral artery: a longitudinal angiographic study. Ann Biomed Eng.

[33] Eto K, Tumenbayar B, Nagashima S, Tazoe F, Miyamoto M, Takahashi M (2010). Distinct association of serum FGF21 or adiponectin levels with clinical parameters in patients with type 2 diabetes. Diabetes Res Clin Pract.

[34] Galman C, Lundasen T, Kharitonenkov A, Bina HA, Eriksson M, Hafstrom I (2008). The circulating metabolic regulator FGF21 is induced by prolonged fasting and PPARalpha activation in man. Cell Metab.

[35] Tyynismaa H, Raivio T, Hakkarainen A, Ortega-Alonso A, Lundbom N, Kaprio J (2011). Liver fat but not other adiposity measures influence circulating FGF21 levels in healthy young adult twins. J Clin Endocrinol Metab.

